# Brain edema progression rate on serial CT predicts neurological outcome after cardiac arrest: a region-specific biomarker of hypoxic-ischemic brain injury

**DOI:** 10.1016/j.resplu.2026.101509

**Published:** 2026-09-29

**Authors:** Nengyuan Xu, Hoikei Cheong, Zhen De, Xintong Li, Yan Wang, Xiangzhu Zeng, Lanfang Du

**Affiliations:** a Emergency Department, Peking University Third Hospital, 49 North Garden Road, Haidian District, Beijing 100191, China; b Department of Radiology, Peking University Third Hospital, 49 North Garden Road, Haidian District, Beijing 100191, China

**Keywords:** Cardiac arrest, Neurological prognostication, Computed tomography, Brain injury, Brain edema

## Abstract

**Objectives:**

To evaluate the prognostic performance of the attenuation change rate on serial brain computed tomography (CT), a novel quantitative marker of temporal cerebral attenuation change, for predicting poor neurological outcome after cardiac arrest (CA).

**Methods:**

This was a retrospective analysis of a prospective cohort. CA patients who underwent cranial CT both within 12 h and between 12 h and one week after return of spontaneous circulation (ROSC) were included. The attenuation change rate, calculated as the HU difference between paired scans divided by the time interval, was measured in 12 predefined brain regions. Neurological outcome was assessed using the Cerebral Performance Category (CPC) at discharge.

**Results:**

Of 53 patients enrolled, 17 (32.1%) had favorable neurological outcomes at discharge. Patients with poor outcomes exhibited significantly greater attenuation decline rates in all gray matter regions except for gray matter at the centrum semiovale level, including the caudate nucleus (−0.01 vs. 0.02 HU/h), putamen (−0.02 vs. 0 HU/h), thalamus (−0.02 vs. 0.01 HU/h), gray matter of the high convexity (−0.02 vs. 0 HU/h), and cerebellar gray matter (−0.02 vs. 0.03 HU/h) (all *P* < 0.01), whereas no statistically significant differences were observed in any of the white matter regions. ROC analysis demonstrated AUCs ranging from 0.725 to 0.778, with cerebellar gray matter achieving the highest AUC (0.778; sensitivity, 75.0%; specificity, 76.5%). At the 100% specificity threshold, the caudate nucleus showed the highest sensitivity for predicting poor outcome (36.1% at a decline rate of −0.040 HU/h).

**Conclusion:**

Serial CT-derived attenuation change rate may serve as an exploratory quantitative marker of cerebral edema progression after cardiac arrest. However, this single-center, small-sample study does not support its use for withdrawal of life-sustaining treatment decisions. Multicenter validation is warranted.

## Highlights

• Poor neurological outcomes were associated with greater attenuation decline in multiple gray matter regions.
• Cerebellar gray matter showed the highest predictive performance (AUC 0.778; sensitivity 75.0%; specificity 76.5%).
• Caudate attenuation decline had the highest sensitivity (36.1%) at 100% specificity.
• Serial CT attenuation change rate may provide a quantitative marker of hypoxic-ischemic brain injury.

## Introduction

Cardiac arrest (CA) is a major public health challenge worldwide, with high rates of mortality and long-term disability among survivors.^1^, ^2^, ^3^ In hospitalized patients who achieve return of spontaneous circulation (ROSC), hypoxic-ischemic brain injury (HIBI) remains the leading cause of death and poor neurological outcomes.^4^, ^5^ Accurate prognostication is therefore essential to avoid an inappropriate withdrawal of life-sustaining treatment (WLST) in patients who still have a chance of a neurologically meaningful recovery and to avoid futile treatment in patients with a severe and irreversible brain injury.^6^, ^7^, ^8^

Brain computed tomography (CT) is widely used to assess the severity of HIBI after CA. Current guidelines from the European Resuscitation Council and the American Heart Association recommend obtaining head CT no earlier than 2 days after CA for neurological prognostication, focusing on qualitative signs of diffuse anoxic injury on a single scan without considering changes over time.^9^, ^10^, ^11^, ^12^ Clinically, the evolution of edema is highly heterogeneous; as demonstrated by Cronberg et al., generalized edema is evident in only 9.6% of early CTs within 24 h, whereas its prevalence increases substantially to 45.5% during days 1–7.^13^ However, many patients also undergo brain CT immediately upon hospital arrival to rule out intracranial hemorrhage, creating an underused opportunity to evaluate the temporal evolution of HIBI. Following CA, global cerebral ischemia triggers both cytotoxic and vasogenic edema. Cytotoxic edema results primarily from energy failure and intracellular water accumulation, whereas vasogenic edema is associated with blood–brain barrier disruption and extracellular fluid accumulation. The relative contributions of these two mechanisms to cerebral edema following CA remain difficult to quantify clinically.^14^, ^15^, ^16^ This progressive brain tissue edema develops over hours to days and represents a key pathophysiological process of HIBI. On non-contrast cranial CT, cytotoxic edema manifests as reduced attenuation in both gray and white matter, with a more pronounced decrease in gray matter.^17^, ^18^ This attenuation change can be quantified by measuring Hounsfield units (HU) in corresponding gray and white matter structures.^19^ Despite its biological and clinical relevance, serial assessment of brain radiodensity changes over time has not been systematically investigated as a prognostic tool.

We hypothesized that the early dynamic rate of HU change on serial CT scans, termed the brain edema rate, may serve as a novel, quantitative, and region-specific neuroimaging biomarker for HIBI. The brain edema rate was calculated as the decline in HU between early and follow-up scans divided by the corresponding time interval. This biomarker may enable more precise tracking of cerebral injury progression. In this study, we aimed to evaluate the prognostic value of the edema rate for predicting poor neurological outcome after CA.

However, the current European Resuscitation Council (ERC) and American Heart Association (AHA) guidelines emphasize that neuroprognostication after CA should be multimodal and should meet very high specificity thresholds before being used for WLST decisions.^20^ The present study is therefore exploratory and hypothesis-generating; it does not aim to propose a stand-alone biomarker for WLST decision-making.

## Methods

### Study population and ethics

We conducted a retrospective analysis of a single-center prospective cohort of adult comatose CA admitted to Peking University Third Hospital between December 2014 and December 2024. The prospective cohort study was approved by the Medical Ethics Committee of Peking University Third Hospital (Approval No. PUTH2022-160-02). Written informed consent was obtained from all patients and/or their legal guardian(s). Patients who underwent cranial CT both within 12 h and between 12 h and one week after ROSC were included. Patients were excluded if they had: (1) a pre-arrest Cerebral Performance Category (CPC) score of 3 or 4; (2) CA of primary neurological etiology (e.g., intracranial hemorrhage); (3) poor-quality brain CT scans that did not allow reliable measurement of gray and white matter attenuation at the region of interest (ROI).

### Measurement of the brain edema rate on CT

In our cardiac arrest center, the initial CT scan is performed as soon as possible after ROSC to rule out primary intracranial causes of cardiac arrest, such as acute stroke or hemorrhage. A follow-up CT scan is typically obtained later, with the primary indication being neuroprognostication to evaluate the evolution of hypoxic-ischemic injury and cerebral edema. This approach is consistent with common practice in our setting, where CT remains one of the most accessible modalities for prognostication in the intensive care unit (ICU) setting.^21^

Non-enhanced CT imaging was performed using a Siemens dual-source flash scanner following standard clinical protocols. Routine scanner quality assurance and calibration were performed according to institutional practice. The two CT examinations for each patient were obtained within one week. All CT scans were evaluated for extensive artifacts by a blinded, board-certified neuroradiologist. Two observers, blinded to clinical outcomes, independently measured cerebral tissue attenuation on axial cranial CT scans using circular ROIs sized 10–15 mm^2^. For each predefined ROI, bilateral measurements were obtained at symmetrical locations on five selected axial slices, and the mean HU across these measurements was used for analysis. Attenuation measurements were obtained at four anatomical regions: (1) basal ganglia, comprising the caudate nucleus, putamen, posterior limb of the internal capsule, genu of the corpus callosum, and thalamus—of which the caudate nucleus, putamen, and thalamus are gray matter structures, whereas the posterior limb of the internal capsule and genu of the corpus callosum are white matter structures; (2) centrum semiovale and high convexity, comprising cerebral cortical gray matter and subcortical white matter; (3) cerebellum, comprising cerebellar gray matter and cerebellar white matter; and (4) pons (which contains both gray and white matter, with white matter predominating), comprising the midbrain and bilateral anterior brainstem regions, with the average of the three ROIs representing the value for this region. For each ROI, the brain edema progression rate—attenuation change rate (ΔHU/*t*)—between paired CT scans was calculated using the following formula:

$$Attenuation\;change\;rate\;\left(\frac{\Delta HU}{t},\frac{\;HU}{h}\right)=\left(HU_{2}-\;HU_{1}\right)\;/\;\left(t_{2}-\;t_{1}\right)$$

where HU_1_ and HU_2_ represent the Hounsfield unit values measured on the initial and follow-up CT scans, respectively, and *t*_1_ and *t*_2_ denote the corresponding time points (in hours) after ROSC. Because a decrease in HU indicates attenuation loss associated with edema progression, negative ΔHU/*t* values represent a decline in attenuation, whereas positive values indicate an increase.

### Clinical end points

Neurological outcome was assessed at hospital discharge by investigators blinded to imaging data using the CPC scale, and dichotomized as favorable (CPC 1–2) or poor (CPC 3–5). The CPC score was independently assigned by two physicians (one emergency physician and one neurologist) from the cardiac arrest multidisciplinary team, based on direct clinical examination and the standard CPC definitions. Any discrepancies between the two raters were resolved by adjudication from a third senior physician. No additional structured checklist or interview instrument was used.

### Statistical analysis

Statistical analyses were performed using SPSS 26.0 and R. Continuous variables were presented as median (IQR; 25th–75th percentile) and compared using the Wilcoxon rank-sum test (Mann–Whitney *U* test). Categorical variables were compared using Pearson's chi-square test or Fisher's exact test, as appropriate. Time to awakening was defined as the interval between ROSC and documented recovery of consciousness. Because patients with unfavorable neurological outcomes did not have documented recovery of consciousness during hospitalization, the analysis of time to awakening was restricted to patients with favorable neurological outcomes who subsequently awakened. Spearman’s rank correlation analysis was used to assess the association between time from ROSC to awakening and the HU decline rate in each of the 12 predefined regions of interest. The mean values of measurements obtained by the two observers were used for all analyses. Interobserver agreement was assessed using the intraclass correlation coefficient (ICC) based on a two-way mixed-effects model with absolute agreement. ICC values were interpreted as follows: <0.20, poor agreement; 0.20–0.39, fair agreement; 0.40–0.59, moderate agreement; 0.60–0.79, strong agreement; and ≥0.80, excellent agreement. Negative ICC values were considered indicative of poor interobserver agreement. To further quantify measurement variability, the mean absolute difference and the standard deviation (SD) of the paired differences between the two observers were calculated for each ROI and CT examination. The standard error of measurement (SEM) was estimated as the SD of the paired differences divided by √2, and the minimum detectable change at the 95% confidence level (MDC95) was calculated as 1.96 × √2 × SEM. These analyses were performed using the original HU measurements obtained independently by the two observers. As an exploratory analysis, the observed longitudinal change in HU (ΔHU) for each ROI was compared with the corresponding measurement-variability reference. For each anatomical region, the larger MDC95 value obtained from the initial and follow-up CT examinations was selected as the more conservative region-specific reference threshold. A ΔHU below −MDC95 was therefore considered to represent a longitudinal attenuation decline exceeding this conservative reference level of measurement variability. This threshold-based analysis was exploratory and was not intended to establish a biological or prognostic cutoff. To assess whether baseline attenuation influenced the association between regional HU decline rate and neurological outcome, binary logistic regression models were constructed for each predefined ROI, with neurological outcome as the dependent variable, HU decline rate as the predictor of interest, and the corresponding baseline HU on the initial CT scan as a covariate. Regression coefficients (*B*) and adjusted *P* values were reported. Receiver operating characteristic (ROC) curve analysis was performed to evaluate the prognostic performance of the attenuation change rate for predicting unfavorable neurological outcome. The area under the ROC curve (AUC) with 95% confidence interval was reported, and optimal cut-off values were determined using the Youden index. For the exploratory comparison with the conventional gray‑to‑white matter ratio (GWR) approach, patients who underwent repeat CT imaging at 48–96 h after ROSC were further analyzed. The prognostic performance of GWR on the second CT scan, regional attenuation change rates, and their combinations was assessed using the AUC with 95% confidence intervals. The DeLong test was performed in R to compare the AUCs of the combined models with GWR alone, and integrated discrimination improvement (IDI) and net reclassification improvement (NRI) were calculated in R to assess the incremental discriminatory and reclassification performance of adding ΔHU/*t* to GWR. Given the limited sample size, this analysis was considered exploratory. All tests were two-tailed, and *P* < 0.05 was considered statistically significant.

## Results

Among the 533 patients in the initial registry, 313 achieved sustained ROSC and constituted the overall ROSC population, while 53 patients met the prespecified eligibility criteria and underwent serial CT examinations and were included in the final study cohort (Fig. 1). Comparison between the overall ROSC population and the final study cohort showed generally small standardized mean differences (SMDs) based on Cohen’s criteria, ranging from <0.01 to 0.35. The largest differences were observed for shockable rhythm (SMD = 0.35), time from arrest to ROSC (SMD = 0.33), targeted temperature management (TTM) (SMD = 0.28), and arrest location (SMD = 0.21), while the remaining variables showed smaller differences. The baseline characteristics of the overall ROSC population and final study cohort are presented in Table 1. The median age was 61.0 (46.0, 75.5) years, and 34 patients (64.2%) were male. Out-of-hospital CA occurred in 30 patients (56.6%). 17 patients (32.1%) presented with a shockable rhythm, and 20 (37.7%) received TTM. The median time from arrest to ROSC was 19.0 (10.0, 34.5) min. The median time from ROSC to the first and second cranial CT was 3.8 (2.2, 6.9) h and 80.9 (70.2, 162.2) h, respectively. The timing of the follow-up CT examination was comparable between outcome groups. The median time from ROSC to the second CT was 72.9 (70.2, 168.0) h in patients with favorable neurological outcomes and 81.0 (62.8, 160.7) h in those with unfavorable outcomes (*P* = 0.939). Five patients (9.43%) had regained consciousness before the second CT examination, indicating that follow-up imaging was not restricted to patients who remained comatose. At hospital discharge, 17 patients (32.1%) had favorable neurological outcomes (CPC 1–2). Compared with those with poor outcomes, patients with favorable outcomes were younger and had higher proportions of out-of-hospital CA, shockable rhythm, and TTM.

**Fig. 1:**
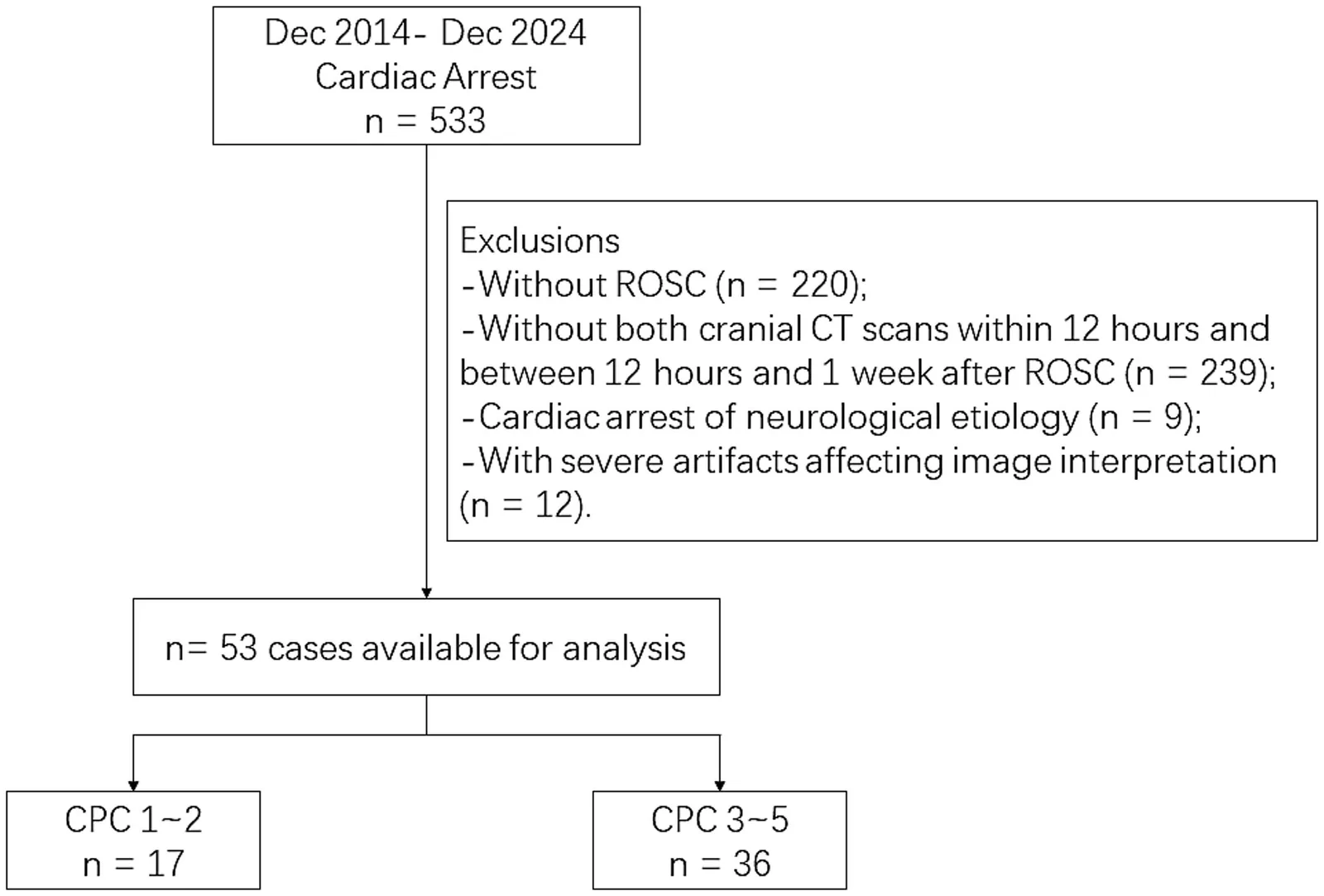
**Study flow chart**. *Abbreviations*: CPC, Cerebral Performance Category; CT, computed tomography; ROSC, return of spontaneous circulation.

**Table 1:** Baseline characteristics of patients in the overall ROSC population and final study cohort.

**Variables**	**Overall ROSC population** **(*n* = 313)**	**Final study cohort** **(*n* = 53)**	**SMD**†	**CPC 1–2** **(*n* = 17)**	**CPC 3–5** **(*n* = 36)**	***P***‡
Age, years median (*p*25, *p*75)	64.5 (50.0, 75.0)	61.0 (46.0, 75.5)	0.16	49.0 (31.0, 67.5)	63.0 (54.3, 79.8)	0.007
Gender, male, *n* (%)	201 (64.2)	34 (64.2)	<0.01	10 (58.8)	24 (66.7)	0.578
Out-of-hospital, *n* (%)	145 (46.3)	30 (56.6)	0.21	13 (76.5)	17 (47.2)	0.045
Witnessed, *n* (%)	301 (96.2)	52 (98.1)	0.11	17 (100.0)	35 (97.2)	1.000
Bystander CPR, *n* (%)	235 (75.1)	44 (83.0)	0.19	14 (82.4)	30 (83.3)	1.000
Arrest to ROSC, minutes median (*p*25, *p*75)	35.0 (14.0, 60.0)	19.0 (10.0, 34.5)	0.33	27.0 (10.0, 34.0)	18.5 (10.5, 33.8)	0.552
Shockable rhythm, *n* (%)	55 (17.6)	17 (32.1)	0.35	10 (58.8)	7 (19.4)	0.004
TTM, *n* (%)	78 (24.9)	20 (37.7)	0.28	11 (64.7)	9 (25.0)	0.005
Time (ROSC to first CT), hours median (*p*25, *p*75)	−	3.8 (2.2, 6.9)	−	3.2 (1.7, 6.1)	4.2 (2.7, 7.1)	0.282
Time (ROSC to second CT), hours median (*p*25, *p*75)	−	80.9 (70.2, 162.2)	−	72.9 (70.2, 168.0)	81.0 (62.8, 160.7)	0.939

*Abbreviations:* ROSC, return of spontaneous circulation; CPC, Cerebral Performance Category; CPR, cardiopulmonary resuscitation; TTM, targeted temperature management; CT, computed tomography.

Data are presented as median (*p*25, *p*75) or *n* (%), as appropriate.

† SMD indicates the standardized mean difference between the overall ROSC population and the final study cohort. According to Cohen’s criteria, an absolute SMD of 0.20, 0.50, and 0.80 represents small, moderate, and large effect sizes, respectively.

‡ *P* value indicates the comparison between CPC 1–2 and CPC 3–5 groups within the final study cohort.

### Time to awakening and its association with HU decline rate

Among the 53 patients included in the serial CT analysis, 17 patients had a favorable neurological outcome and regained consciousness during hospitalization, whereas no patients with an unfavorable neurological outcome had documented recovery of consciousness during hospitalization. Among the 17 patients with favorable neurological outcomes, the median time from ROSC to awakening was 112.95 (63.23, 202.07) h. Five patients (9.43% of the entire cohort and 29.4% of patients with favorable neurological outcomes) had awakened before the second CT examination.

Spearman’s rank correlation analysis showed no statistically significant monotonic association between time from ROSC to awakening and the HU decline rate in any of the 12 predefined regions of interest. The Spearman correlation coefficients ranged from −0.432 to 0.302, and all corresponding *P* values were >0.05 (Supplementary Table S1).

### Interobserver agreement analysis

Two blinded observers independently measured HU values in all ROIs on the obtained CT scans. Interobserver agreement is summarized in Supplementary Table S2. All ICC analyses yielded *P* < 0.05. However, for cerebellar white matter HU measurements on CT scans obtained after 12 h post-ROSC, interobserver reliability was poor (ICC = 0.446; 95% CI, −0.084 to 0.709). For all other measurements, interobserver agreement was moderate to excellent.

### Quantification of interobserver measurement variability

The quantitative assessment of interobserver measurement variability is presented in Supplementary Table S3. Among the gray-matter regions of primary interest, the mean absolute interobserver differences ranged from 1.19 to 2.27 HU across the two CT examinations. The corresponding SDs of the paired differences ranged from 1.66 to 3.04 HU, and the estimated MDC95 values ranged from 3.26 to 5.97 HU. The smallest mean absolute difference was observed for the follow-up caudate nucleus measurements (1.19 HU), whereas the largest was observed for the initial cerebellar gray matter measurements (2.27 HU). These findings indicate that individual HU measurements were subject to several HU of measurement variability, which was considered when interpreting the relatively small ΔHU/*t* values.

### Exploratory analysis using MDC95 as a reference for measurement variability

For the initial head CT measurements, the MDC95 values were 4.41 HU for the caudate nucleus, 3.97 HU for the putamen, 4.19 HU for the thalamus, 5.34 HU for gray matter of the high convexity, and 5.97 HU for cerebellar gray matter. For the follow-up head CT measurements, the corresponding MDC95 values were 3.26 HU, 3.92 HU, 3.98 HU, 4.62 HU, and 4.59 HU, respectively. For the exploratory threshold-based analysis, the larger MDC95 value between the initial and follow-up CT examinations was used for each anatomical region as a conservative reference for measurement variability.

Using these region-specific conservative thresholds, 9/53 (17.0%) patients had a caudate nucleus ΔHU below −MDC95, 7/53 (13.2%) had a putamen ΔHU below −MDC95, 9/53 (17.0%) had a thalamus ΔHU below −MDC95, 3/53 (5.7%) had a gray matter of the high convexity ΔHU below −MDC95, and 2/53 (3.8%) had a cerebellar gray matter ΔHU below −MDC95. These cases were markedly concentrated among patients with poor neurological outcomes. All 9 patients with a caudate nucleus ΔHU below −MDC95 had poor neurological outcomes, whereas none of the patients with favorable outcomes met this criterion. For the putamen, 6/7 patients (85.7%) with a ΔHU below −MDC95 had poor outcomes and 1/7 (14.3%) had a favorable outcome. For the thalamus, 7/9 patients (77.8%) with a ΔHU below −MDC95 had poor outcomes and 2/9 (22.2%) had favorable outcomes. All patients with a ΔHU below −MDC95 in the gray matter of the high convexity (3/3) and cerebellar gray matter (2/2) had poor neurological outcomes. Thus, attenuation declines exceeding the conservative measurement-variability reference were predominantly observed among patients with poor neurological outcomes across all five gray matter regions.

### CT attenuation values on serial scans

Comparisons of CT attenuation values between the two groups, stratified by neurological outcome at discharge, on both the first (within 12 h after ROSC) and the second (between 12 h and 1 week after ROSC) scans are presented in Table 2. On both CT scans, patients with poor neurological outcomes had significantly lower attenuation values in the caudate nucleus, putamen, and gray matter of the high convexity than those with favorable outcomes. In addition, the decrease in HU values from the initial to the follow‑up scan in the aforementioned three ROIs was significantly greater in patients with poor neurological outcomes than in those with favorable outcomes. For the thalamus and cerebellar gray matter, no statistically significant differences in HU values were observed between the favorable and poor neurological outcome groups on the initial CT scan. On the follow‑up CT scan, however, HU values in both regions were significantly lower in the poor‑outcome group compared with the favorable‑outcome group. Furthermore, the ΔHU values (defined as HU_follow‑up_ − HU_initial_) were significantly lower in the poor‑outcome group than in the favorable‑outcome group for both the thalamus (−2.0 vs. 1.3 HU; *P* = 0.006) and the cerebellar gray matter (−1.8 vs. 2.0 HU; *P* = 0.001). In contrast, no statistically significant differences were observed in the posterior limb of the internal capsule, genu of the corpus callosum, cerebellar white matter, or pons, regardless of outcome group or scan timing.

**Table 2:** CT attenuation values (HU) in predefined brain regions on initial and follow-up scans, stratified by neurological outcome at discharge.

**ROIs**	**CT scan within 12 h after ROSC**			**CT scan between 12 h and 1 week after ROSC**			ΔHU*		
	**CPC 1–2** ***n* = 17**	**CPC 3–5** ***n* = 36**	***P***	**CPC 1–2** ***n* = 17**	**CPC 3–5** ***n* = 36**	***P***	**CPC 1–2** ***n* = 17**	**CPC 3–5** ***n* = 36**	***P***
**Basal ganglia**									
Caudate nucleus	36.3 (34.7, 38.0)	35.0 (33.3, 36.1)	0.032	37.3 (35.5, 39.2)	34.5 (29.4, 35.5)	<0.001	1.5 (−0.9, 3.1)	−1.1 (−4.4, 0.9)	0.005
Putamen	36.1 (34.5, 38.3)	35.1 (33.5, 36.3)	0.040	36.5 (35.4, 37.7)	33.0 (30.9, 35.4)	<0.001	−0.2 (−1.5, 2.7)	−1.4 (−3.2, 0.4)	0.023
Posterior limb of internal capsule	28.7 (27.2, 29.9)	28.3 (27.7, 29.0)	0.446	28.3 (27.9, 29.8)	28.2 (26.9, 29.1)	0.179	0.5 (−1.4, 1.4)	0.0 (−1.3, 1.2)	0.675
Genu of corpus callosum	29.6 (28.0, 31.3)	29.7 (28.7, 31.2)	0.724	30.0 (28.7, 31.4)	29.5 (28.1, 30.9)	0.265	−0.1 (−1.0, 2.5)	0.1 (−2.4, 1.1)	0.219
Thalamus	34.6 (33.9, 36.6)	35.1 (33.4, 35.8)	0.647	36.5 (34.3, 37.1)	32.9 (29.7, 35.2)	0.002	1.3 (−1.0, 2.8)	−2.0 (−3.3, -0.3)	0.006
**Centrum semiovale and high convexity**									
Gray matter at the centrum semiovale level	35.5 (34.0, 37.3)	34.3 (32.6, 35.5)	0.021	34.8 (33.7, 37.1)	33.2 (31.1, 34.9)	0.004	−0.9 (−2.6, 2.1)	−0.4 (−2.0, 0.8)	0.762
Gray matter of the high convexity	37.1 (35.3, 38.2)	35.4 (33.5, 36.5)	0.006	38.2 (35.7, 38.9)	33.4 (32.2, 35.4)	<0.001	0.5 (−0.9, 1.6)	−1.3 (−3.4, 0.5)	0.008
White matter at the centrum semiovale level	29.8 (28.9, 30.7)	28.7 (27.5, 30.4)	0.059	29.7 (28.4, 31.4)	28.3 (27.1, 29.2)	0.007	0.1 (−1.3, 1.4)	−0.7 (−2.1, 1.0)	0.320
White matter of the high convexity	31.9 (30.1, 32.4)	30.9 (29.1, 31.5)	0.153	32.0 (29.7, 32.9)	29.9 (28.0, 31.0)	0.007	0.7 (−1.4, 2.2)	−0.5 (−2.4, 0.8)	0.120
**Cerebellum**									
Cerebellar gray matter	43.0 (40.7, 44.7)	43.4 (41.0, 44.4)	0.804	43.4 (42.5, 46.3)	41.7 (40.2, 43.5)	0.001	2.0 (−0.7, 2.7)	−1.8 (−2.8, 0.0)	0.001
Cerebellar white matter	32.4 (30.5, 34.1)	32.9 (30.8, 35.5)	0.312	32.4 (30.9, 34.0)	32.7 (30.9, 34.3)	0.864	0.2 (−2.0, 2.8)	−0.7 (−2.5, 1.6)	0.523
**Pons**	31.5 (29.6, 33.0)	30.6 (29.0, 31.7)	0.245	32.0 (29.6, 33.5)	31.2 (28.6, 32.7)	0.195	1.4 (−3.2, 4.4)	0.2 (−2.5, 2.2)	0.703

*Abbreviations:* CT, computed tomography; HU, Hounsfield unit; ROI, region of interest; ROSC, return of spontaneous circulation; CPC, Cerebral Performance Category.

* The difference in CT attenuation values between the second scan (obtained between 12 h and 1 week after ROSC) and the first scan (obtained within 12 h after ROSC). Values are presented as median (*p*25, *p*75).

Representative CT images and individual-level paired HU measurements are shown in Supplementary Figs. S1 and S2. Supplementary Fig. S1 illustrates the change in gray matter attenuation between the initial CT obtained 6.08 h after ROSC and the follow-up CT obtained at 48.15 h in a patient with an unfavorable neurological outcome, with HU values annotated for the caudate nucleus, putamen, thalamus, and cerebellum. Supplementary Fig. S2 presents paired scatterplots of initial versus follow-up HU values for the predefined gray matter regions, stratified by neurological outcome. The identity line represents unchanged attenuation, whereas values below the line indicate a decrease in HU between the two examinations. A greater downward shift was visually apparent in several gray matter regions among patients with unfavorable outcomes, particularly in the caudate nucleus and putamen, whereas greater overlap between outcome groups was observed in other regions. To further visualize regional changes in CT attenuation, mean HU values for the 12 predefined brain regions at the initial and follow-up CT examinations were plotted and stratified by neurological outcome (Supplementary Fig. S3). The plots provide a complementary group-level visualization of the direction and magnitude of attenuation changes over time.

### The rate of attenuation decline

Among the 12 ROIs assessed, patients with poor neurological outcomes exhibited significantly greater attenuation decline rates in all gray matter regions except for gray matter at the centrum semiovale level, including the caudate nucleus (−0.01 vs. 0.02 HU/h; *P* = 0.005), putamen (−0.02 vs. 0 HU/h; *P* = 0.009), thalamus (−0.02 vs. 0.01 HU/h; *P* = 0.004), gray matter of the high convexity (−0.02 vs. 0 HU/h; *P* = 0.009), and cerebellar gray matter (−0.02 vs. 0.03 HU/h; *P* = 0.001), whereas no statistically significant differences were observed in any of the white matter regions (Table 3).

**Table 3:** Regional attenuation change rates (HU/h) and their associations with neurological outcome after cardiac arrest.

**ROIs**	**CPC 1–2** ***n* = 17**	**CPC 3–5** ***n* = 36**	**Unadjusted *P***	**Regression coefficient (*B*)**	**Adjusted *P***
**Basal ganglia**					
Caudate nucleus	0.02 (0, 0.04)	−0.01 (−0.05, 0.01)	0.005	−0.700	0.006
Putamen	0 (−0.01, 0.03)	−0.02 (−0.05, 0)	0.009	−0.614	0.008
Posterior limb of internal capsule	0.01 (−0.01, 0.01)	0 (−0.01, 0.02)	0.481	−0.311	0.229
Genu of corpus callosum	0 (−0.01, 0.03)	0 (−0.02, 0.01)	0.261	−0.002	0.993
Thalamus	0.01 (−0.01, 0.04)	−0.02 (−0.05, 0)	0.004	−0.268	0.097
**Centrum semiovale and high convexity**					
Gray matter at the centrum semiovale level	−0.01 (−0.02, 0.03)	0 (−0.02, 0.01)	0.634	−0.428	0.009
Gray matter of the high convexity	0 (−0.01, 0.02)	−0.02 (−0.03, 0)	0.009	−0.966	0.001
White matter at the centrum semiovale level	0 (−0.01, 0.01)	−0.01 (−0.02, 0.01)	0.208	−0.432	0.036
White matter of the high convexity	0.01 (−0.01, 0.02)	−0.01 (−0.02, 0.01)	0.109	−0.450	0.029
**Cerebellum**					
Cerebellar gray matter	0.03 (0, 0.04)	−0.02 (−0.03, 0)	0.001	−0.107	0.482
Cerebellar white matter	0 (−0.02, 0.02)	−0.01 (−0.03, 0.02)	0.391	0.144	0.403
**Pons**	0.02 (−0.02, 0.05)	0 (−0.02, 0.03)	0.493	−0.148	0.244

*Abbreviations:* HU, Hounsfield unit; ROI, region of interest; CPC, Cerebral Performance Category.

Values are presented as median (*p*25, *p*75), unless otherwise indicated. Unadjusted *P* values were obtained from comparisons of regional HU decline rates between patients with favorable (CPC 1–2) and unfavorable (CPC 3–5) neurological outcomes. Regression coefficients (*B*) and adjusted *P* values were derived from binary logistic regression models assessing the association between regional HU decline rate and neurological outcome, with adjustment for the corresponding baseline regional HU measured on the initial CT scan.

After adjustment for the corresponding baseline regional HU, the associations between regional HU decline rate and neurological outcome remained statistically significant for the caudate nucleus (*B* = −0.700, *P* = 0.006), putamen (*B* = −0.614, *P* = 0.008), gray matter at the centrum semiovale level (*B* = −0.428, *P* = 0.009), gray matter of the high convexity (*B* = −0.966, *P* = 0.001), white matter at the centrum semiovale level (*B* = −0.432, *P* = 0.036), and white matter of the high convexity (*B* = −0.450, *P* = 0.029). The associations were not statistically significant after adjustment for the thalamus (*B* = −0.268, *P* = 0.097), cerebellar gray matter (*B* = −0.107, *P* = 0.482), cerebellar white matter (*B* = 0.144, *P* = 0.403), posterior limb of internal capsule (*B* = −0.311, *P* = 0.229), genu of corpus callosum (*B* = −0.002, *P* = 0.993), or pons (*B* = −0.148, *P* = 0.244). Although no significant between-group differences were observed in white-matter ΔHU/*t* in the unadjusted analyses, baseline-adjusted models showed statistically significant associations in white matter regions at the centrum semiovale and high convexity.

A sensitivity analysis was performed by restricting the second CT examination to 13–72 h after ROSC to assess the robustness of the findings to variation in follow-up CT timing. In the sensitivity analysis, 26 patients were included (CPC 1–2, *n* = 9; CPC 3–5, *n* = 17). The results were broadly consistent with those of the primary analysis. ΔHU/*t* remained significantly different between outcome groups in the caudate nucleus (*P* = 0.010), putamen (*P* = 0.033), thalamus (*P* = 0.019), and cerebellar gray matter (*P* = 0.043). Gray matter of the high convexity showed a similar direction of association, although the difference did not reach statistical significance (*P* = 0.056). The detailed results are presented in Supplementary Table S4.

### The performance of regional attenuation change rates for poor neurological outcome

The predictive performance of regional attenuation decline rates for poor neurological outcome is summarized in Table 4 and Fig. 2. Using thresholds derived from the Youden index, the cerebellar gray matter exhibited the highest AUC (0.778), with a sensitivity of 75.0% and specificity of 76.5%. This was followed by the thalamus (AUC, 0.745; sensitivity, 77.8%; specificity, 70.6%), caudate nucleus (AUC, 0.743; sensitivity, 44.4%; specificity, 94.1%), gray matter of the high convexity (AUC, 0.725; sensitivity, 69.4%; specificity, 70.6%), and putamen (AUC, 0.725; sensitivity, 61.1%; specificity, 82.4%). At the 100% specificity threshold, the caudate nucleus achieved the highest sensitivity (36.1% at −0.040 HU/h), while sensitivities for the remaining regions ranged from 11.1% to 30.6%.

**Table 4:** Receiver operating characteristic (ROC) analysis for poor outcome prediction using regional attenuation change rates (HU/h).

**ROIs**	**AUC [95% CI]**	**Maximum Youden's index**				Specificity = 100%*		***P***
		**Youden's index**	**Sensitivity**	**Specificity**	**Cut-off value**	**Sensitivity**	**Cut-off value**	
Caudate nucleus	0.743 [0.607–0.880]	0.386	0.444	0.941	−0.016	0.361	−0.040	0.005
Putamen	0.725 [0.581–0.870]	0.435	0.611	0.824	−0.012	0.139	−0.093	0.009
Thalamus	0.745 [0.601–0.889]	0.484	0.778	0.706	−0.002	0.111	−0.099	0.004
Gray matter of the high convexity	0.725 [0.589–0.862]	0.400	0.694	0.706	−0.005	0.306	−0.025	0.009
Cerebellar gray matter	0.778 [0.640–0.916]	0.515	0.750	0.765	−0.001	0.111	−0.062	0.001

*Abbreviations:* HU, Hounsfield unit; ROI, region of interest.

* Cut-off value and corresponding sensitivity at 100% specificity.

**Fig. 2:**
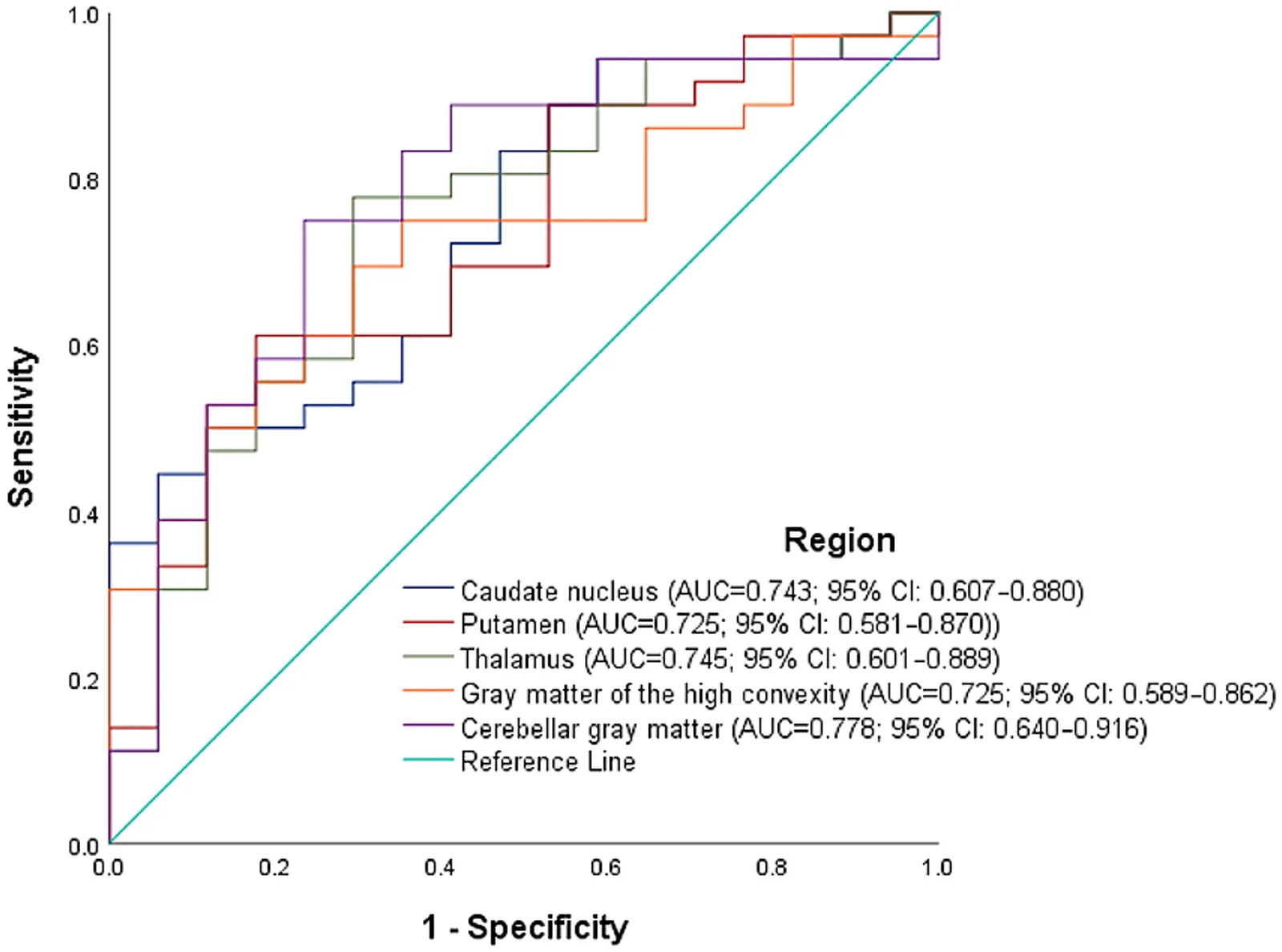
**Receiver operating characteristic (ROC) curves of attenuation change rates in five brain regions for predicting poor neurological outcomes following cardiac arrest**.

To further evaluate the relationship between the ΔHU/*t* and conventional GWR, an exploratory analysis was performed in the 28 patients who underwent repeat CT imaging at 48–96 h after ROSC. The prognostic performance of GWR alone and in combination with regional ΔHU/*t* is summarized in Supplementary Table S5, with the definitions and calculation of regional GWR provided in Supplementary Table S6. In the basal ganglia, GWR demonstrated excellent discrimination (AUC = 0.964), and the addition of caudate nucleus ΔHU/*t* resulted in only a small numerical increase in AUC to 0.983 (incremental AUC = 0.019), without significant improvement by the DeLong test (*P* = 0.197), IDI (*P* = 0.415), or NRI (*P* = 0.147). In contrast, the combination of cerebellum GWR and cerebellar gray matter ΔHU/*t* increased the AUC from 0.839 to 0.917 (incremental AUC = 0.078), with significant improvements in IDI (*P* = 0.031) and NRI (*P* = 0.005), although the DeLong test was not significant (*P* = 0.934). The combination of GWR at the centrum semiovale and high convexity and gray matter of the high convexity ΔHU/*t* also showed a numerical increase in AUC from 0.769 to 0.839 (incremental AUC = 0.070), but the DeLong test, IDI, and NRI did not demonstrate statistically significant improvement. These findings indicate that the potential incremental contribution of ΔHU/*t* may vary across anatomical regions and according to the baseline discriminatory performance of GWR.

## Discussion

This study provides evidence that CA patients with poor neurological outcomes exhibit a significantly more rapid decline in CT attenuation across multiple gray matter regions, including the caudate nucleus, putamen, thalamus, high convexity gray matter, and cerebellar gray matter, whereas no such differences are observed in any white matter region. Among the regions assessed, cerebellar gray matter attenuation decline offers the best overall discriminatory performance for predicting unfavorable outcomes, while the caudate nucleus provides the highest sensitivity at 100% specificity. These findings suggest that the gray matter edema progression rate, as reflected by serial CT attenuation decline, can serve as an objective early imaging marker for neuroprognostication following CA.

Brain edema is a hallmark of hypoxic-ischemic encephalopathy after cardiac arrest, arising from two distinct but overlapping pathophysiological processes.^15^, ^22^ The primary injury involves energy failure, which drives intracellular sodium accumulation and cytotoxic edema, while the secondary injury is characterized by reperfusion-mediated extravasation of intravascular water across a disrupted blood–brain barrier, leading to vasogenic edema.^23^ Kenda et al. quantified regional net water uptake (NWU) on serial CT as a direct measure of brain edema severity and reported that an NWU threshold exceeding 8% in the putamen or caudate nucleus predicted poor outcome with 100% specificity.^14^ This study provides preliminary evidence that the rate of cerebral CT attenuation change may have prognostic value after CA. While traditional GWR cut-offs (e.g., <1.10 or <1.20) have demonstrated high specificity, their sensitivity has been limited and variable.^19^, ^24^, ^25^ A dynamic decline in caudate nucleus attenuation may provide information on evolving cerebral injury that is not captured by a single-time-point GWR measurement.

The greater attenuation decline observed in gray matter than in white matter is consistent with the greater vulnerability of metabolically active neuronal tissue to hypoxic-ischemic injury. Gray matter, composed of neuronal cell bodies with high metabolic demands and dense synaptic activity, is more susceptible to energy failure and subsequent cytotoxic edema. In contrast, white matter, consisting primarily of myelinated axons with lower metabolic requirements, can tolerate hypoxia longer before structural changes become evident on CT.^26^ Many previous original studies and meta‑analyses have confirmed that decreased GWR, driven largely by gray matter hypoattenuation, is strongly associated with poor outcomes, highlighting gray matter integrity as a critical prognostic determinant.^27^, ^28^, ^29^, ^30^ Building on this, Gramespacher et al demonstrated that machine learning‑based gray matter analysis of early post‑arrest CT imaging provided significant prognostic discrimination between favorable and unfavorable neurological outcomes, outperforming the conventional GWR.^31^

Importantly, the associations between regional HU decline rates and neurological outcome remained statistically significant after adjustment for baseline regional HU in several regions, including caudate nucleus, putamen, and gray matter of the high convexity. These findings suggest that the observed associations in these regions were not solely attributable to differences in baseline attenuation or a potential floor effect. However, the associations were attenuated and no longer statistically significant in some regions, including the thalamus and cerebellar gray matter, indicating that baseline attenuation may influence the magnitude of the observed association in a region-specific manner. These discrepant findings between unadjusted group comparisons and baseline-adjusted models should be interpreted cautiously, particularly given the small sample size and exploratory nature of the analyses.

In our study, the caudate nucleus demonstrated the highest sensitivity (36.1%) at a decline rate threshold of −0.040 HU/h while maintaining 100% specificity, indicating that it is particularly sensitive for detecting early hypoxic‑ischemic brain injury after CA. The caudate nucleus, together with the putamen, represents one of the key gray matter structures at the basal ganglia level that is especially vulnerable to ischemia due to its high metabolic demand.^32^, ^33^ They are also clearly distinguishable and less affected by partial volume effects in comparison to the cerebral cortex, making them ideal for attenuation measurements. Previous studies have consistently shown that decreased attenuation in the caudate nucleus on non-contrast CT correlates with poor neurological outcomes.^30^, ^34^, ^35^ Hrdlicka et al. demonstrated that GWR values in the caudate nucleus within a narrow range of 1.18–1.30 effectively separated patients with favorable from those with unfavorable outcomes, achieving a sensitivity of 78% and a specificity of 85%.^36^ Furthermore, Kenda et al. reported that NWU >8% in the caudate nucleus predicted poor outcome with 100% specificity and 43% sensitivity,^14^ underscoring the prognostic relevance of this region.

Our finding that cerebellar gray matter assessment achieved the highest predictive accuracy for unfavorable outcomes (AUC 0.778, sensitivity 75.0%, specificity 76.5%) underscores the cerebellum as a prognostically relevant structure that has been historically underemphasized in post CA neuroprognostication. Traditional views hold that cerebellar Purkinje cells exhibit relative resilience to hypoxia–ischemia and animal studies showed less severe Purkinje cell degeneration compared with hippocampal pyramidal neurons, likely due to protective mechanisms involving cerebellar astrocytes and antioxidant defenses.^37^, ^38^ Nevertheless, ischemia–reperfusion injury still occurs when the insult is sufficiently severe. Importantly, oxcarbazepine protects Purkinje cells by attenuating oxidative stress,^39^ and xenon combined with hypothermia significantly preserves gray matter volume in the cerebellar cortex,^40^ highlighting the cerebellum as a tractable target for neuroprotection and corroborating its sensitivity to both ischemic injury and therapeutic response. Furthermore, Arctaedius et al proposed a standardized semi-quantitative Cardiac Arrest Brain Ischemia (CABI) score that divides the brain into 12 predefined territories, each scored 0–3 for ischemic severity, with the cerebellum and brainstem evaluated as independent regions alongside three cortical areas and two deep gray matter nuclei; this system achieved AUCs of 0.87–0.92.^41^ These findings suggest that the cerebellum may represent a potentially independent component of neuroprognostication, distinct from the cerebral cortex.

In the present study, the attenuation change rate in the gray matter of the high convexity differed significantly between patients with favorable and poor neurological outcomes, whereas no significant difference was observed in the gray matter at the centrum semiovale level. The gray matter at the centrum semiovale level is located closer to the boundary zone between the cortical branches of the anterior cerebral artery (ACA) and the middle cerebral artery (MCA)—namely, the anterior watershed region.^42^ In the setting of global hypoperfusion following cardiac arrest, watershed zones are among the most vulnerable areas and the earliest to sustain ischemic injury, with nearly universal involvement.^43^ This widespread damage may dilute between-group differences, as both favorable- and poor-outcome patients exhibit substantial attenuation decline in this region. In contrast, the gray matter of the high convexity has a relatively more stable blood supply, and the extent of its injury correlates more directly with the overall severity of ischemia, thereby enabling more sensitive differentiation of neurological outcomes. Furthermore, from a morphometric perspective, the cortical gray matter of the high convexity is relatively thicker, facilitating ROI placement with minimal contamination from adjacent white matter or sulcal cerebrospinal fluid (CSF). By contrast, the cortical gray matter at the centrum semiovale level tends to be thinner, making it more susceptible to partial volume effects from neighboring white matter and CSF signal, which may increase measurement variability and reduce the reliability of attenuation measurements.^44^ These anatomical and morphometric differences explain why these two cortical gray matter regions, despite both being classified as gray matter, demonstrate distinctly different prognostic values. This finding also suggests that in future neuroprognostic studies, careful selection of ROIs—with avoidance of watershed zones—is warranted to minimize the “floor effect” that may attenuate prognostic discrimination.

Furthermore, it is important to distinguish the proposed ΔHU/*t* from the traditional GWR. GWR is a well-established static marker reflecting the relative attenuation difference between gray and white matter at a specific time point,^45^ whereas ΔHU/*t* is a dynamic marker capturing the temporal trajectory and rate of cerebral injury progression. Thus, the two measures may provide complementary rather than redundant information. In particular, a relatively preserved GWR at an earlier stage may coexist with a rapid decline in gray matter attenuation, potentially providing information on evolving cerebral edema before a conventional GWR threshold is reached.^24^ To explore the potential incremental prognostic value of ΔHU/*t* beyond GWR, we performed an exploratory analysis in 28 patients with repeat CT scans at 48–96 h after ROSC. The incremental contribution of ΔHU/*t* varied across regions. In the basal ganglia, where late GWR already demonstrated excellent prognostic performance (AUC = 0.964), adding caudate nucleus ΔHU/*t* resulted in only a small numerical increase in AUC (0.964–0.983), with no significant improvement by DeLong, IDI, or NRI testing. This may partly reflect a ceiling effect of the high-performing basal ganglia GWR. In contrast, the combination of cerebellum GWR and cerebellar gray matter ΔHU/*t* showed a greater numerical increase in AUC (0.839–0.917), with significant improvements in IDI and NRI, although not by the DeLong test. These findings suggest that the potential complementary value of ΔHU/*t* may be more apparent in regions where static GWR has lower discriminatory performance. However, given the small sample size and exploratory nature of this analysis, these findings should be considered hypothesis-generating. Therefore, ΔHU/*t* should not currently be considered a replacement for GWR, but rather a complementary imaging marker within a multimodal prognostication framework, particularly when serial neuroimaging is available. Larger prospective studies are needed to determine whether incorporating dynamic attenuation trajectories into established GWR-based prognostic strategies provides clinically meaningful incremental value.

An important methodological consideration is the relatively small numerical magnitude of the attenuation decline rates. Because ΔHU/*t* is calculated by dividing the cumulative attenuation change by the interval between paired CT examinations, hourly values may be numerically small even when the absolute attenuation change over the follow-up interval is several HU. Thus, a small ΔHU/*t* should not be interpreted as indicating that the underlying attenuation change is negligible. Quantitative CT attenuation measurements are subject to variability related to scanner characteristics, acquisition parameters, ROI placement, and observer-related factors. Previous studies have demonstrated inter-scanner variability in brain HU measurements, influencing both absolute HU values and the GWR.^46^ Similarly, previous methodological work has shown that reproducible CT number measurements can be obtained with standardized ROI placement and repeated measurements, while HU values may still vary between scanners.^47^ In the present study, both CT examinations for each patient were performed on the same scanner using standardized acquisition protocols, with routine quality assurance and calibration according to institutional practice, and paired examinations obtained within one week. These measures substantially reduced potential inter-scanner variability and short-term scanner drift, although residual technical variability cannot be fully excluded. ROI-related variability was minimized by predefined anatomical landmarks, standardized ROI sizes (10–15 mm^2^), blinded independent measurements by two observers, and averaging across five axial slices. The observed mean absolute interobserver differences in the gray-matter regions of primary interest ranged from 1.19 to 2.27 HU, and the estimated MDC95 values ranged from 3.26 to 5.97 HU. These findings provide a quantitative estimate of interobserver variability in our dataset and indicate that individual-level attenuation changes of only a few HU should be interpreted cautiously. To further examine whether the observed longitudinal attenuation changes were large relative to the measurement variability, we performed an exploratory analysis using the MDC95 as a reference. For each anatomical region, the larger MDC95 value obtained from the initial and follow-up CT examinations was selected as a conservative, region-specific reference threshold. Using these thresholds, 9 patients (17.0%) had a caudate nucleus ΔHU below −MDC95, 7 (13.2%) had a putamen ΔHU below −MDC95, 9 (17.0%) had a thalamus ΔHU below −MDC95, 3 (5.7%) had a gray matter of the high convexity ΔHU below −MDC95, and 2 (3.8%) had a cerebellar gray matter ΔHU below −MDC95. Notably, these attenuation declines were markedly concentrated among patients with poor neurological outcomes. All 9 patients with a caudate nucleus ΔHU below −MDC95 had poor neurological outcomes, whereas none of the patients with favorable outcomes met this criterion. Similarly, 6 of 7 patients (85.7%) with a putamen ΔHU below −MDC95 and 7 of 9 patients (77.8%) with a thalamus ΔHU below −MDC95 had poor neurological outcomes. Importantly, all patients meeting this criterion in the gray matter of the high convexity (3/3) and cerebellar gray matter (2/2) had poor neurological outcomes. These findings provide additional descriptive evidence that more pronounced longitudinal attenuation declines relative to the observed interobserver measurement variability were predominantly concentrated among patients with poor neurological outcomes. Nevertheless, these threshold-based findings should be interpreted cautiously. The MDC95 values were derived from interobserver measurement variability for individual CT examinations rather than from longitudinal test–retest measurements of ΔHU. Therefore, the MDC95 should not be interpreted as a validated biological or prognostic cutoff. Rather, it was used as a conservative reference to contextualize the magnitude of longitudinal HU changes relative to the measurement variability observed in our dataset. Importantly, we do not assume that every individual ΔHU exceeds this measurement variability. The additional analysis showed that the measurement variability of individual HU measurements was several HU in the gray matter regions. Accordingly, individual-level attenuation changes of only a few HU should be interpreted with caution, whereas the observed concentration of changes exceeding the conservative reference level in patients with poor neurological outcomes provides supportive, albeit exploratory, evidence for the observed group-level association. Partial-volume effects cannot be completely eliminated by standard CT acquisition and remain a potential source of variability, particularly in relatively thin cortical gray-matter regions. In this study, their impact was minimized through standardized ROI placement and averaging across multiple slices. Nevertheless, residual partial-volume effects may have contributed to measurement variability, particularly in cortical regions, and should be considered when interpreting small attenuation changes. The use of a 5-mm slice thickness alone cannot exclude such effects. There is currently no universally applicable measurement-error threshold for gray-matter HU across different CT scanners and acquisition protocols. Quantitative head CT measurements are influenced by imaging parameters and scanner characteristics, and standardization of acquisition protocols is therefore important for longitudinal and multicenter quantitative analyses.^48^ Accordingly, this study should not be interpreted as establishing that every observed ΔHU/*t* value exceeds a predefined measurement-error threshold. Rather, the robustness of ΔHU/*t* in this cohort is supported by standardized imaging conditions, blinded repeated measurements, moderate-to-excellent interobserver agreement, quantitative assessment of measurement variability, the observed concentration of larger attenuation declines in patients with poor neurological outcomes, significant between-group differences in several gray matter regions, and broadly consistent sensitivity analyses. Residual measurement variability remains an important limitation, and the threshold-based analysis should be considered exploratory. Further prospective multicenter studies using standardized CT acquisition and quantitative quality-control procedures are warranted to establish the external reproducibility and clinically meaningful change thresholds of ΔHU/*t*. Such studies may also help determine whether measurement-error-adjusted changes in regional gray matter attenuation provide reproducible prognostic information across different scanners, acquisition protocols, and patient populations.

This study has several limitations. First, as a single-center study with a relatively small sample size (*n* = 53), the generalizability of the findings may be limited. The limited number of patients, particularly those with favorable neurological outcomes (*n* = 17), increased the risk of overfitting in the ROC analysis and may have reduced statistical power to detect more subtle differences or to adjust for potential confounders in multivariable models. Larger multicenter cohort studies are warranted to validate the thresholds proposed in this study and to confirm the robustness of the attenuation change rate as a prognostic biomarker. Second, the inclusion criterion requiring patients to undergo two cranial CT scans (one within 12 h after ROSC and another between 12 h and one week after ROSC) may have introduced significant selection bias. In addition, the timing of the follow-up CT was not standardized. Because cerebral edema evolves non-linearly over time, ΔHU/*t* represents an average rate of attenuation change between two CT examinations and may not fully capture phase-specific changes in edema progression. Although the sensitivity analysis restricted to patients undergoing follow-up CT between 13 and 72 h after ROSC yielded broadly consistent findings, residual effects of heterogeneous CT timing cannot be excluded. Prospective studies with standardized serial CT time points are needed to better characterize the temporal trajectory of attenuation changes. Patients who were too clinically unstable to undergo a second CT scan, or those who underwent WLST based on early prognostication before completing the follow-up scan, were necessarily excluded from this study. This may have resulted in a study cohort that was relatively “healthier” compared to the overall post-cardiac arrest population, thereby affecting the generalizability of the prognostic performance estimates and limiting the applicability of the findings to a broader population of cardiac arrest patients. The timing of neurological recovery may also have influenced the likelihood of obtaining a follow-up CT and therefore represents a potential source of selection bias. In our cohort, 17 patients with favorable neurological outcomes regained consciousness during hospitalization, with a median time to awakening of 112.95 h after ROSC. Five patients (9.43%) had awakened before the second CT examination. Furthermore, no statistically significant monotonic association was observed between time to awakening and regional HU decline rates, with Spearman’s correlation coefficients ranging from −0.432 to 0.302 across the 12 predefined regions. These findings do not suggest a clear association between the timing of neurological recovery and the observed rate of attenuation change. Nevertheless, because the timing and indication for follow-up CT were determined by clinical practice rather than a prespecified research protocol, selection bias cannot be excluded. Patients with early neurological recovery or an unequivocally poor clinical trajectory may have been less likely to undergo serial CT imaging. To further assess the representativeness of the study sample, we compared the overall ROSC population with the final study cohort. Most baseline characteristics differed minimally; however, small to moderate differences were observed in arrest location, time from arrest to ROSC, shockable rhythm, and TTM use. These differences likely reflect the clinical profile of patients who survived to ROSC and remained sufficiently stable to undergo serial CT examinations. Accordingly, selection bias cannot be excluded, and the prognostic performance estimates derived from the final cohort may not be directly generalizable to the broader population of patients achieving ROSC. Third, follow-up was conducted only until hospital discharge, which may be insufficient for evaluating long-term neurological outcomes in cardiac arrest patients. Future studies should extend the follow-up period to at least one year. Despite these limitations, our findings provide preliminary evidence that the density attenuation change rate in specific brain regions may serve as a predictor of neurological outcomes in cardiac arrest patients.

## Conclusion

This study demonstrated that cardiac arrest patients with unfavorable neurological outcomes showed significantly greater CT attenuation decline rates in gray matter regions, including the caudate nucleus, putamen, thalamus, high convexity, and cerebellum, but not in white matter. Cerebellar gray matter attenuation decline showed the best overall discrimination for unfavorable neurological outcomes, while caudate nucleus attenuation decline achieved the highest sensitivity at 100% specificity. These findings suggest that regional gray matter attenuation decline may serve as a potential imaging marker for neurological prognostication after cardiac arrest and warrant further validation in larger, multicenter studies.

## CRediT authorship contribution statement

**Nengyuan Xu:** Writing – review & editing, Writing – original draft, Visualization, Methodology, Investigation, Formal analysis, Data curation, Conceptualization. **Hoikei Cheong:** Investigation, Formal analysis, Data curation. **Zhen De:** Investigation, Data curation. **Xintong Li:** Investigation, Data curation. **Yan Wang:** Resources, Formal analysis. **Xiangzhu Zeng:** Resources, Formal analysis. **Lanfang Du:** Writing – review & editing, Supervision, Resources, Project administration, Funding acquisition, Data curation, Conceptualization.

## Funding

This work was supported by The National Natural Science Foundation of China (Grant No. 62273009), and the Emergency-Critical Care Delirium Cohort BYSYDL2025023.

## Declaration of competing interest

We declare that we have no known competing financial interests and have no other relevant declarations.

## Figures and Tables

**Supplementary Fig. S1:**
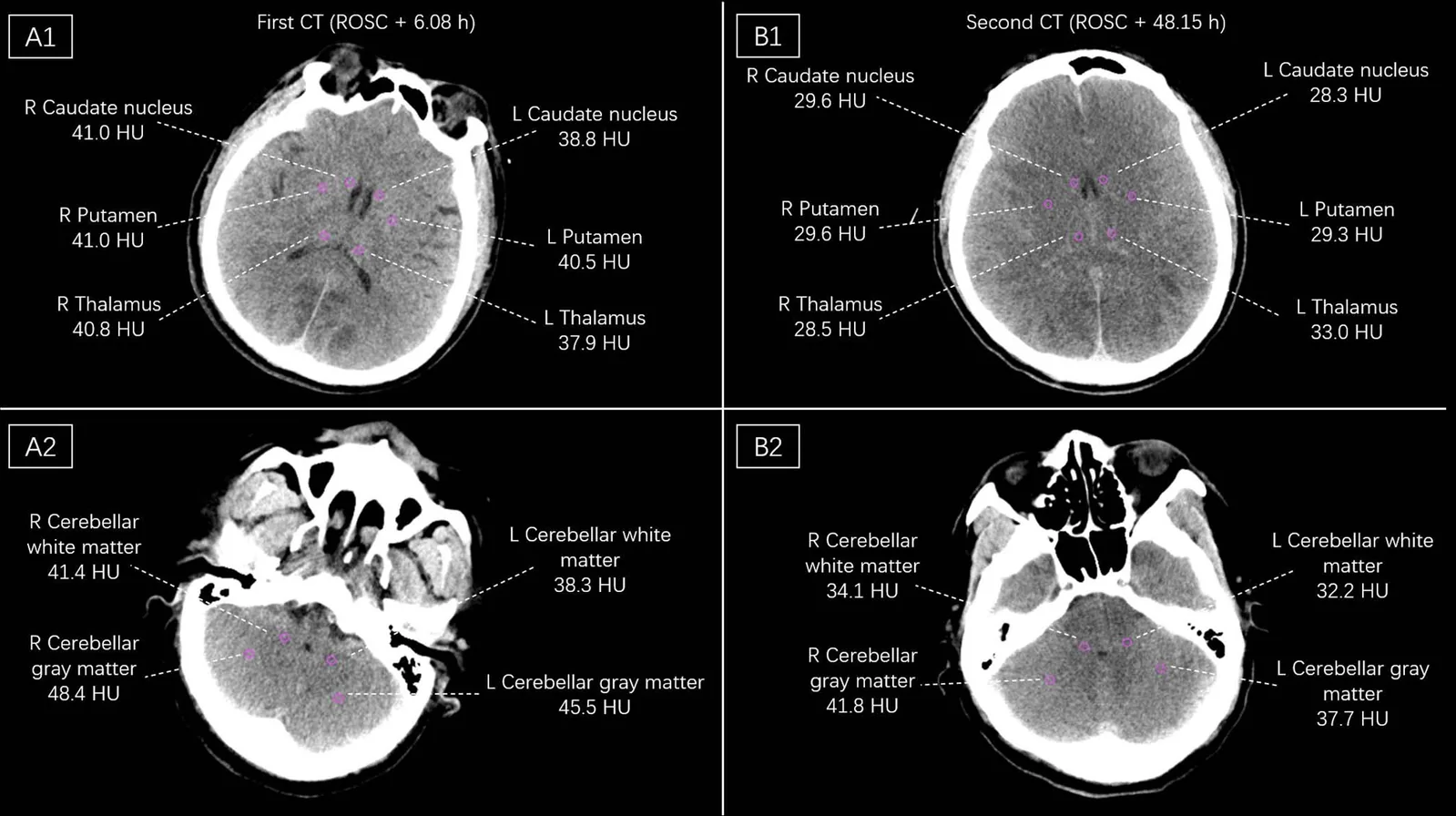
**Representative serial non-contrast head CT images demonstrating gray matter attenuation decline in a patient with an unfavorable neurological outcome.** (A) Initial (6.08 h post-ROSC) and (B) follow-up (48.15 h post-ROSC) CT images from a patient with a poor neurological outcome, with annotated HU values in the gray matter regions of basal ganglia and cerebellum illustrating the interval attenuation decline. A1 and B1 show the basal ganglia, and A2 and B2 show the cerebellum. Circular ROIs with annotated HU values are placed in the bilateral caudate nucleus, putamen, and thalamus (A1, B1) and in the bilateral cerebellar gray and white matter (A2, B2). An interval decline in HU values is visually apparent in several gray matter regions in this representative case. *Abbreviations:* CT, computed tomography; ROSC, return of spontaneous circulation; HU, Hounsfield unit; L, left; R, right; ROI, region of interest.

**Supplementary Fig. S2:**
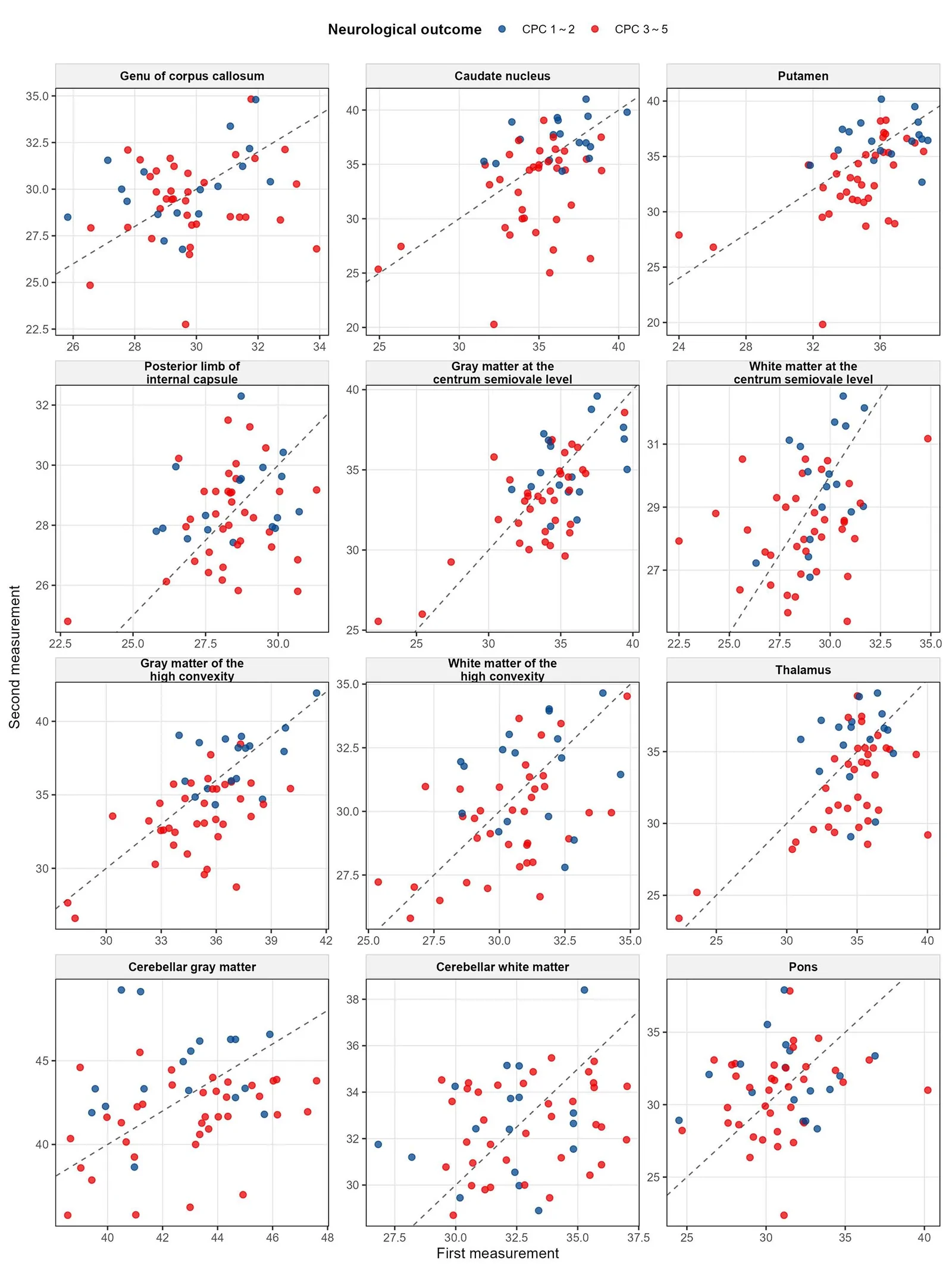
**Paired scatterplots of initial and follow-up CT attenuation values in 12 predefined brain regions, stratified by neurological outcome at discharge.** Each panel displays the HU value on the first CT scan (*x*-axis) versus the second CT scan (*y*-axis) for an individual patient. Blue points represent patients with favorable neurological outcomes (CPC 1–2), and red points represent patients with poor neurological outcomes (CPC 3–5). The dashed diagonal line indicates the line of identity (*y* = *x*); points below the line indicate a decrease in HU values from the first to the second scan, whereas points above the line indicate an increase. *Abbreviations:* CPC, Cerebral Performance Category; CT, computed tomography; HU, Hounsfield unit.

**Supplementary Fig. S3:**
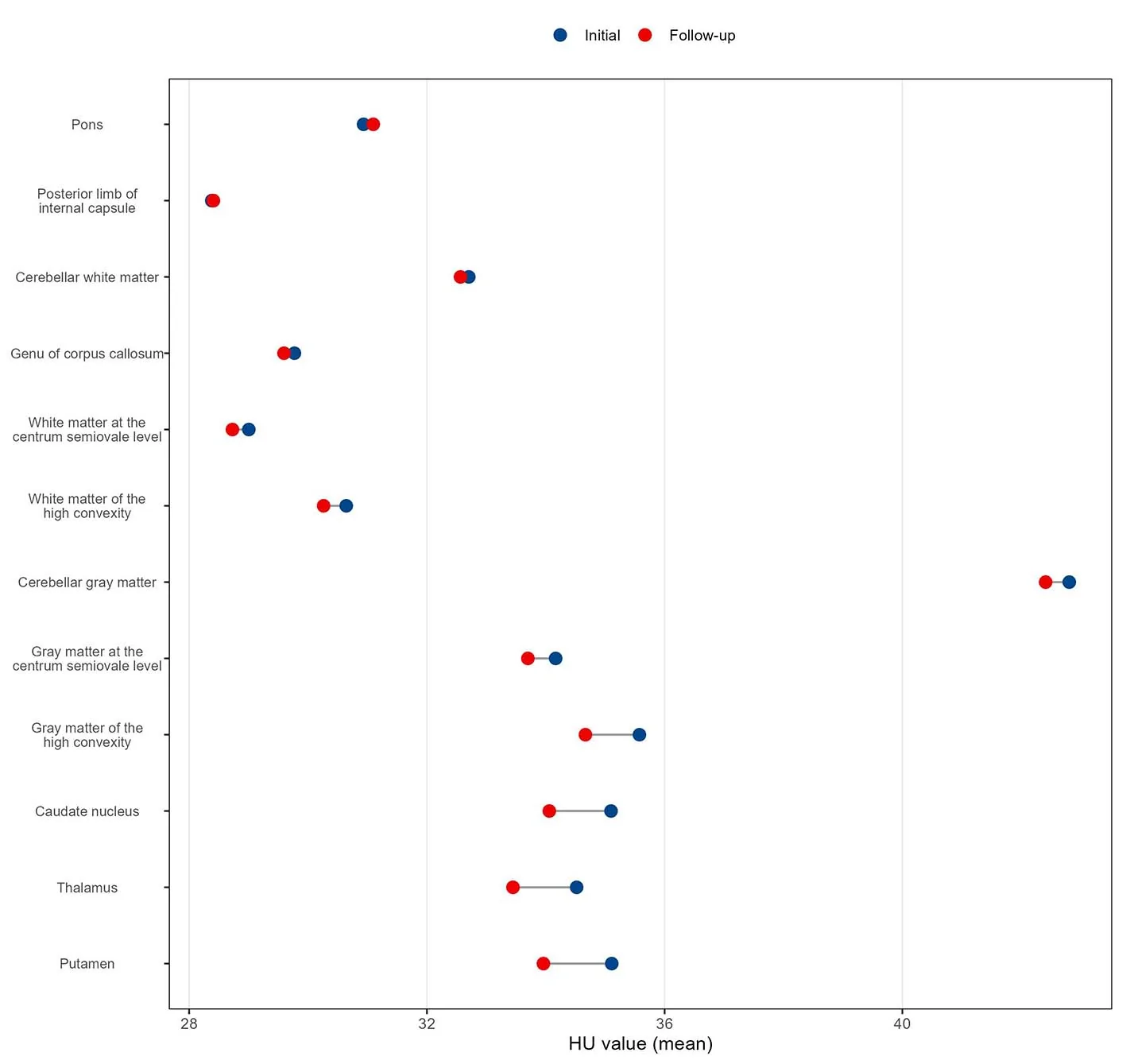
**Mean CT attenuation values in 12 predefined brain regions on initial and follow-up scans, stratified by neurological outcome at discharge.** Blue points represent mean HU values on the initial CT scan, and red points represent mean HU values on the follow-up CT scan. Horizontal lines connect the initial and follow-up values within each region, illustrating the direction and magnitude of attenuation change over time. *Abbreviations:* CT, computed tomography; HU, Hounsfield unit.
