# Supplementary material for: Brain edema progression rate on serial CT predicts neurological outcome after cardiac arrest: a region-specific biomarker of hypoxic-ischemic brain injury
Source: Resusc Plus. 2026 Sep 29;32:101509. doi: 10.1016/j.resplu.2026.101509 (PMC13682381; doi:10.1016/j.resplu.2026.101509)
Supplement: Supplementary file 1 [file mmc4.docx]

| **Supplementary Table S1. Exploratory Spearman correlation analysis between time to awakening and regional HU decline rates.** | | | | |
| --- | --- | --- | --- | --- |
| ROIs | | | Spearman's ρ | *P* |
| Basal ganglia | | | | |
|  | Caudate nucleus | -0.113 | | 0.666 |
|  | Putamen | -0.133 | | 0.612 |
|  | Posterior limb of internal capsule | -0.147 | | 0.572 |
|  | Genu of corpus callosum | 0.079 | | 0.764 |
|  | Thalamus | -0.432 | | 0.083 |
| Centrum semiovale and high convexity | | | | |
|  | Gray matter at the centrum semiovale level | 0.088 | | 0.736 |
|  | Gray matter of the high convexity | 0.172 | | 0.509 |
|  | White matter at the centrum semiovale level | -0.064 | | 0.808 |
|  | White matter of the high convexity | 0.034 | | 0.896 |
| Cerebellum | | | | |
|  | Cerebellar gray matter | -0.425 | | 0.089 |
|  | Cerebellar white matter | -0.115 | | 0.659 |
| Pons | | | 0.302 | 0.238 |
| This analysis was exploratory and was performed to assess the potential association between time to awakening and regional HU decline rates. Spearman’s ρ denotes Spearman’s rank correlation coefficient.  *Abbreviations*: HU, Hounsfield unit; ROI, region of interest. | | | | |

| **Supplementary Table S2. Interobserver agreement for cerebral tissue attenuation measurements.** | | | |  |
| --- | --- | --- | --- | --- |
| ROIs | | ICC [95% CI] | |  |
|  |  | CT scan within 12 hours after ROSC | CT scan between 12 hours and 1 week after ROSC |  |
| Basal ganglia | | | |  |
|  | Caudate nucleus | 0.824 [0.681-0.901] | 0.956 [0.906-0.977] | |
|  | Putamen | 0.858 [0.753-0.919] | 0.913 [0.828-0.953] | |
|  | Posterior limb of internal capsule | 0.819 [0.688-0.895] | 0.756 [0.576-0.859] | |
|  | Genu of corpus callosum | 0.536 [0.209-0.729] | 0.633 [0.334-0.794] | |
|  | Thalamus | 0.865 [0.763-0.923] | 0.919 [0.860-0.953] | |
| Centrum semiovale and high convexity | | | |  |
|  | Gray matter at the centrum semiovale level | 0.916 [0.854-0.951] | 0.851 [0.741-0.914] | |
|  | Gray matter of the high convexity | 0.715 [0.508-0.835] | 0.860 [0.758-0.919] | |
|  | White matter at the centrum semiovale level | 0.617 [0.336-0.779] | 0.673 [0.218-0.843] | |
|  | White matter of the high convexity | 0.404 [0.002-0.649] | 0.752 [0.313-0.888] | |
| Cerebellum | | | |  |
|  | Cerebellar gray matter | 0.571 [0.267-0.750] | 0.809 [0.607-0.900] | |
|  | Cerebellar white matter | 0.544 [0.199-0.739] | 0.446 [-0.084-0.709] | |
| Pons | | 0.737 [0.542-0.848] | 0.914 [0.851-0.950] |  |
| All *P*＜0.05.  *Abbreviations*: ROI, region of interest; ICC, intraclass correlation coefficient; CT, computed tomography; ROSC, post-return of spontaneous circulation. | | | |  |

| **Supplementary Table S3. Quantification of interobserver measurement variability for cerebral tissue attenuation (HU).** | | | | | |
| --- | --- | --- | --- | --- | --- |
| ROIs | CT examination | Mean absolute difference | SD of differences | SEM | MDC95 |
| Caudate nucleus | Initial | 1.50 | 2.25 | 1.59 | 4.41 |
|  | Follow-up | 1.19 | 1.66 | 1.18 | 3.26 |
| Putamen | Initial | 1.33 | 2.03 | 1.43 | 3.97 |
|  | Follow-up | 1.72 | 2.00 | 1.41 | 3.92 |
| Thalamus | Initial | 1.52 | 2.14 | 1.51 | 4.19 |
|  | Follow-up | 1.55 | 2.03 | 1.44 | 3.98 |
| Gray matter of the high convexity | Initial | 2.03 | 2.72 | 1.93 | 5.34 |
|  | Follow-up | 1.66 | 2.36 | 1.67 | 4.62 |
| Cerebellar gray matter | Initial | 2.27 | 3.04 | 2.15 | 5.97 |
|  | Follow-up | 2.09 | 2.34 | 1.66 | 4.59 |
| SEM was calculated as SD of the paired interobserver differences/√2. MDC95 was calculated as 1.96 × √2 × SEM.  *Abbreviations*: ROI, region of interest; CT, computed tomography; HU, Hounsfield unit; SD, standard deviation; SEM, standard error of measurement; MDC95, minimum detectable change at the 95% confidence level. | | | | | |

| **Supplementary Table S4.** **Sensitivity analysis of regional attenuation change rates (HU/h) according to neurological outcome.** | | | | | |  |
| --- | --- | --- | --- | --- | --- | --- |
| ROIs | | | CPC 1-2  n=9 | CPC 3-5  n=17 | *P* |  |
| Basal ganglia | | | | | |  |
|  | Caudate nucleus | 0.04(0.01,0.06) | | -0.02(-0.13,0.02) | 0.010 | |
|  | Putamen | 0.03(-0.03,0.05) | | -0.03(-0.08,0.01) | 0.033 | |
|  | Posterior limb of internal capsule | 0.01(-0.01,0.04) | | 0.01(-0.04,0.03) | 0.500 | |
|  | Genu of corpus callosum | 0.03(-0.02,0.05) | | 0(-0.07,0.03) | 0.319 | |
|  | Thalamus | 0.03(-0.02,0.06) | | -0.05(-0.10,-0.01) | 0.019 | |
| Centrum semiovale and high convexity | | | | | |  |
|  | Gray matter at the centrum semiovale level | 0.03(-0.01,0.04) | | 0(-0.06,0.04) | 0.374 | |
|  | Gray matter of the high convexity | 0.01(-0.01,0.05) | | -0.02(-0.05,0.02) | 0.056 | |
|  | White matter at the centrum semiovale level | 0.01(-0.02,0.03) | | -0.02(-0.06,0.04) | 0.269 | |
|  | White matter of the high convexity | 0.01(-0.02,0.05) | | -0.01(-0.04,0.03) | 0.205 | |
| Cerebellum | | | | | |  |
|  | Cerebellar gray matter | 0.03(0.02,0.04) | | -0.02(-0.06,0.02) | 0.043 | |
|  | Cerebellar white matter | 0(-0.02,0.04) | | -0.01(-0.06,0.02) | 0.247 | |
| Pons | | | 0.03(-0.06,0.07) | -0.02(-0.06,0.04) | 0.346 |  |
| This sensitivity analysis included 26 patients whose second CT examination was performed between 13 and 72 hours after ROSC.  *Abbreviations*: HU, Hounsfield unit; ROI, region of interest; CPC, Cerebral Performance Category; CT, computed tomography; ROSC, return of spontaneous circulation. | | | | | |  |

| **Supplementary Table S5. Exploratory analysis of the incremental prognostic performance of brain edema progression rate beyond GWR.** | | | | | | | | |
| --- | --- | --- | --- | --- | --- | --- | --- | --- |
| ROIs | | AUC [95% CI] | *P* | Incremental AUC | DeLong *P* | IDI *P* | NRI *P* | |
| BG-GWR | | 0.964 [0.898,1.000] | ＜0.001 | - | - | - | - | |
| Caudate ΔHU/t | | 0.861 [0.712,1.000] | 0.002 | - | - | - | - | |
| Putamen ΔHU/t | | 0.756 [0.558,0.953] | 0.027 | - | - | - | - | |
| Thalamus ΔHU/t | | 0.806 [0.619,0.992] | 0.008 | - | - | - | - | |
|  | BG-GWR + Caudate ΔHU/t | 0.983 [0.945,1.000] | ＜0.001 | 0.019 | 0.197 | 0.415 | 0.147 | |
|  | BG-GWR + Putamen ΔHU/t | 0.961 [0.896,1.000] | ＜0.001 | -0.003 | 0.047 | 0.937 | 0.253 | |
|  | BG-GWR + Thalamus ΔHU/t | 0.972 [0.914,1.000] | ＜0.001 | 0.008 | 0.110 | 0.531 | 0.377 | |
| CS-HC-GWR | | 0.769 [0.597,0.942] | 0.020 | - | - | - | - | |
| HC-GM ΔHU/t | | 0.806 [0.642,0.969] | 0.008 | - | - | - | - | |
|  | CS-HC-GWR + HC-GM ΔHU/t | 0.839 [0.694,0.984] | 0.003 | 0.070 | 0.720 | 0.149 | 0.268 | |
| CB-GWR | | 0.839 [0.687,0.990] | 0.003 | - | - | - | - | |
| CB-GM ΔHU/t | | 0.850 [0.669,1.000] | 0.003 | - | - | - | - | |
|  | CB-GWR + CB-GM ΔHU/t | 0.917 [0.812,1.000] | ＜0.001 | 0.078 | 0.934 | 0.031 | 0.005 | |
| The analysis included 28 patients who underwent a second CT scan at 48-96 hours after return of spontaneous circulation. AUCs are presented with 95% confidence intervals. *P* values in the AUC column indicate the statistical significance of the corresponding AUC. Incremental AUC represents the difference in AUC between the combined model and GWR alone. The DeLong test was used to compare the AUCs of the combined model and GWR alone, while IDI and NRI were used to assess incremental discrimination and reclassification, respectively. *P* < 0.05 was considered statistically significant.  ΔHU/t, brain edema Rate (HU/h) = (HU _follow-up_ – HU _initial_) / (t _follow-up_ – t _initial_).  *Abbreviations*: ROI, region of interest; GWR, gray‑to‑white matter ratio; HU, Hounsfield unit; AUC, area under the receiver operating characteristic curve; IDI, integrated discrimination improvement; NRI, net reclassification improvement; BG, basal ganglia; CS-HC, centrum semiovale and high convexity; HC-GM, gray matter of the high convexity; CB, cerebellum; CB-GM, cerebellar gray matter; CT, computed tomography. | | | | | | | |  |

| **Supplementary Table S6. Definition and calculation of regional gray-to-white matter ratios.** | |
| --- | --- |
| ROIs | GWR calculation |
| Basal ganglia | (Caudate nucleus + Putamen) / (Posterior limb of internal capsule + Genu of corpus callosum) |
| Centrum semiovale and high convexity | (Gray matter at the centrum semiovale level + Gray matter of the high convexity) / (White matter at the centrum semiovale level + White matter of the high convexity) |
| Cerebellum | Cerebellar gray matter / Cerebellar white matter |
| *Abbreviations*: ROI, region of interest; GWR, gray‑to‑white matter ratio. | |
